# Dietary Non-Esterified Oleic Acid Decreases the Jejunal Levels of Anorectic *N*-Acylethanolamines

**DOI:** 10.1371/journal.pone.0100365

**Published:** 2014-06-24

**Authors:** Thi Ai Diep, Andreas N. Madsen, Sandra Krogh-Hansen, Marwa Al-Shahwani, Laila Al-Sabagh, Birgitte Holst, Harald S. Hansen

**Affiliations:** 1 Department of Drug Design & Pharmacology, Faculty of Health and Medical Sciences, University of Copenhagen, Copenhagen, Denmark; 2 Department of Neuroscience and Pharmacology, Faculty of Health and Medical Sciences, University of Copenhagen, Copenhagen, Denmark; University of East Anglia, United Kingdom

## Abstract

**Background and Aims:**

Oleoylethanolamide and several other *N*-acylethanolamines (NAEs), e.g. linoleoylethanolamide and palmitoylethanolamide, have anorectic properties in rats, and prolonged intake of a high-fat diet decreases the levels of the anorectic NAEs in jejunum. Jejunal anorectic NAEs are thought to add to the control of food intake via activation of PPARalpha and the vagus nerve. The fat-induced decrease may explain part of the hyperphagic effect of high-fat diets. In the present study, we investigated 1) whether the reduced levels of anorectic NAEs were reversible in rats, 2) whether mice respond to dietary fat (olive oil) by reducing levels of anorectic NAEs, and 3) whether dietary non-esterified oleic acid also can decrease levels of anorectic NAEs in mice. We are searching for the fat sensor in the intestine, which mediates the decreased levels of anorectic NAEs.

**Methods:**

Male rats and mice were fed diets high (45 energy% fat) in either triacylglycerol or free fatty acids for 7–14 days, and jejunal NAE and *N*-acylphosphatidylethanolamine (NAPE) levels were determined by liquid-chromatography mass spectrometry.

**Results:**

In rats, reduced levels of anorectic NAEs could be reversed after 3 days from changing the diet from high-fat to chow. Corresponding NAPE levels tended to show the same changes. In mice, jejunal levels of anorectic NAEs were also reduced when fed a high-fat diet. In addition, we found that non-esterified oleic acid were also able to reduce levels of anorectic NAEs in mice.

**Conclusions:**

These results suggest that the down-regulation of the jejunal level of anorectic NAEs by dietary fat is not restricted to rats, and that the fatty acid component oleic acid, in dietary olive oil may be sufficient to mediate this regulation. Thus, a fatty acid sensor may mediate this effect of dietary fat.

## Introduction

*N*-acylethanolamines (NAEs) are a group of lipids composed of ethanolamine coupled to a fatty acid group via an amide bond. They may have various biological functions including appetite regulation, and anti-inflammatory, analgesic and neuromodulatory effects [1]–[7]. Intraperitoneal (ip) injection of oleoylethanolamide (OEA), palmitoylethanolamide (PEA), and linoleoylethanolamide (LEA) reduced food intake in rats [8], [9], and increasing endogenous NAE levels by transiently overexpressing a NAE-forming enzyme in rat small intestine resulted in transiently reduction of food intake [10]. These three NAEs (OEA, LEA, PEA), which account for the bulk of NAEs in the jejunum, are considered to be anorectic, which together may contribute in regulating food intake [11]. Jejunal levels of anorectic NAEs in rats are regulated by the dietary status of the animal, where fasting reduces and refeeding normalizes the levels, respectively [12], [13], and where seven days of high fat diet (HFD) also reduced the intestinal levels of anorectic NAEs [9], [14]. Jejunal levels of anandamide, which also is a NAE, seems to be regulated independently of the anorectic NAEs [11], [13], [15].

We have previously shown that an isocaloric HFD, where the energy density is equal to regular chow (12.5 kJ/g), but having a high fat content (45 energy percentage (E%) fat) reduced jejunal levels of anorectic NAEs, indicating the presence of a sensor in the small intestine, which senses dietary fat, and subsequently decreases the levels of anorectic NAEs in the jejunum [9]. NAEs are formed from the precursor molecules, *N*-acylphosphatidylethanolamines (NAPEs) by several enzymatic pathways including hydrolysis by *N*-acylphosphatidylethanolamine-specific phospholipase D (NAPE-PLD) [16], [17], and it has been shown that during fasting-refeeding, the levels of the individual NAE species are regulated by the levels of their corresponding NAPE species [13]. Thus, it is interesting to know whether jejunal NAPE levels also are decreased by dietary fat. Gillum *et al* [18] has reported that dietary fat increases the release of 16:0-NAPE from the intestine to the vascular system and that this NAPE may function as an anorectic hormone. However, more recent research has not supported a specific anorectic effect of 16:0-NAPE [17], [19].

Dietary fat (mostly triacylglycerol) is upon digestion hydrolyzed in the *sn1* and *sn3* position by a combination of lingual/gastric lipase and pancreas lipase into 2-monoacylglycerol (2-MG) and fatty acids [20], and one of these two metabolites may be responsible for stimulating the fat sensor, which mediates the fat-induced decrease in jejunal levels of anorectic NAEs. Several receptors have recently been identified in the gastrointestinal tract, which respond to the 2-MG and fatty acids, respectively. These include GPR119, which may be stimulated by 2-MG [21], [22], and GPR40 [23], [24], GPR120 [25], and CD36 [26], which may be stimulated by long-chain fatty acids. HFDs are known to induce obesity in experimental animals [27], [28] and in humans [29], and by identifying the dysregulation involved in the metabolic system, we could develop better tools in preventing obesity. Our hypothesis is that the reduction in jejunal levels of anorectic NAEs by intake of HFDs may contribute to this over-consumption of fat calories, and by identifying the dietary component and the mechanism by which it is sensed in the intestine, we will be one step closer to understand the mechanisms triggering obesity by HFDs.

## Methods

### Chemicals and drugs

Chloroform, methanol, ethyl acetate, diethyl ether and hexane were obtained from Merck chemicals (Darmstadt, Germany) and all had purities >99.0%. The chloroform and methanol were free from PEA contaminants [30]. Deuterium-labeled NAEs (^2^H_4_-OEA, ^2^H_4_-PEA, ^2^H_4_-LEA) were purchased from Cayman Chemicals. *Streptomyces chromofuscus* phospholipase D (PLD) was from Calbiochem (Darmstadt, Germany).

### Ethics Statement

All animal studies were approved by the Animal Experimentation Inspectorate of the Danish Ministry of Justice, nr 2009-561-1631 and nr 2009-561-1622.

### Animals, handling and care

Male Sprague-Dawley rats approx. 300 g (Taconic M &B, Lille Skensved, Denmark) and male C57BL/6J approx. 23 g mice (Charles River, Sulzfeld, Germany) were used for the animal studies. The animals were maintained at a 12-h light-dark cycle (06:00/18:00) in temperature (20°C–22°C) and humidity (50–60%) controlled rooms, with free access to standard chow (Altromin 1314F (pellets) or 1311 (powdered), Lage, Germany) and tap water unless otherwise stated. All animals were acclimatized for at least 4 days and 12 h/24 h (onset of dark period) prior to the initiation of the feeding experiment, the mice/rats, respectively, were fasted, but with free access to tap water. All animals had access to food and water throughout the entire study and they were not fasted before sacrifice.

### Diets

All diets were made from powdered chow (12.5 kJ/g; Altromin 1311, Lage, Germany). For olive oil-HFD the fat E% was increased, by adding, olive oil (Santagata Luigi srl, Genova, Italy) to the diet. For the oleic acid-HFD diet, oleic acid (>97%) (VWR BDH Prolabo, Herlev, Denmark) was used as fat source.

For both types of high-fat diets (olive oil and oleic acid-diets) olive oil/oleic acid were mixed into the diet to reach 45E% from fat (19.5 kJ/g). All diets were stored in cold conditions (+5°C) and were changed every second day.

### Rat refeeding study

Fifty-six male Sprague-Dawley rats (approx. 300 g) were housed individually and randomized into 7 groups (n = 8): a control and an olive oil-HFD group, plus 5 groups fed with olive oil-HFD for 7 days and subsequently re-fed for 1,3,5,7,and 14 days, respectively, with regular chow. After the feeding period, the animals were killed by CO_2_/O_2_ anesthesia followed by cervical decapitation and the jejunums were collected and analyzed for NAE content as described by Diep *et al* [9].

### Time study

Twenty-four C57Bl6J mice were housed 4 in each cage, and randomly assigned into 3 groups (n = 8). The control group was fed standard chow for 7 days, whereas the two experimental groups were fed for 3 and 7 days, respectively with olive oil-HFD diet. After the feeding period, the animals were killed by cervical decapitation and the jejunums were isolated and rinsed in ice cold saline as described by Diep *et al* [9]. The excised tissue was snap frozen on crushed dry ice and stored at −80°C until further analysis for NAE content.

### Free fatty acid feeding study

Thirty male C57Bl6 mice were housed individually (TSE LabMaster system, Bad Homburg, Germany), and were as described in Wellner *et al* [19] randomly assigned into 4 groups (n = 7–8): a control group fed with regular chow; a positive control group fed with olive oil-HFD, a group fed with oleic acid-HFD, and a group, which was fed with olive oil-HFD, but was pair-fed to the oleic acid-HFD group. The pair-feeding was done automatically. In the oleic acid-HFD group food intake per hour was measured, and subsequently the pair-fed group was allowed to eat the average amount of food as eaten by the oleic acid-HFD group the previous hour. The animals were fed for 7 days.

After the feeding period, the mice were killed and the jejunums were isolated and rinsed with ice-cold saline as described in Diep *et al* [9]. The excised tissue was snap frozen on crushed dry ice and stored at −80°C until further analysis for NAE and NAPE.

### Sample preparation for NAE and NAPE analysis

All jejunal samples were analyzed for NAE and NAPE content. NAE were analyzed as described by Artmann *et al* [14] with slight modifications. In short, the tissues were homogenized with deuterium-labeled NAEs together with *N*-nonadecanoylphosphatidylethanolamine (19:0-NAPE) as internal standard for NAE and NAPE analysis, respectively. 19:0-NAPE was synthesized as described by Petersen *et al* [31] using the fatty acid nonodecanoic acid (Sigma Aldrich, St Louis, MO, USA). Tissue homogenates were extracted and the NAE and NAPE fractions were separated via SPE silica (Strata, Phenomenex, Værløse, Denmark) solid phase extraction, where NAE fractions were eluted out of the columns using a two-step procedure. First 10 ml 3% MeOH in CHCl_3_ were added to the columns and subsequently 5 ml 5% MeOH in CHCl_3_ were added to the columns. Subsequently, NAPE was eluted out of the columns by addition of 4 ml 25% MeOH in CHCl_3_. The NAPE fractions were afterwards hydrolyzed by PLD using a modified procedure of the one described by Schmid *et al* [32]. In brief, the evaporated NAPE fractions were incubated with 1 ml diethyl ether, 15 µl CaCl_2_ and 2,5 U PLD for 1 hour at 40°C, shaking. After incubation the hydrolyzed NAPEs (now NAEs) were extracted. NAEs were analyzed by LCMS (Hewlett-Packard, Palo Alto, CA, USA) as described previously [14].

### Indirect calorimetry and fat mass determination

Indirect calorimetry was measured in a 16-chamber system (TSE LabMaster System, Bad Homburg, Germany). The mice were housed individually in the chambers for at least 5 days prior to the study start. Food intake, oxygen consumption, respiratory exchange ratio (RER) and activity were measured individually for each mouse for 7 consecutively days.

Fat and lean mass was determined in live non-anesthetized mice using quantitative magnetic resonance imaging (MRI) EchoMRI (4-in-1 Echo Medical Systems, Houston, TX, USA).

### Statistics

Lipid data are presented as mean ± S.E.M. One-way ANOVA with Holm-Bonferroni's multiple comparison correction was used for analysis of NAE and NAPE data. Fischer's PLSD post hoc test was applied to test for differences between the groups. *P*-values <0.05 were considered statistically significant.

## Results

### Chow re-feeding restores jejunal levels of anorectic NAEs in rats

**Figure 1:**
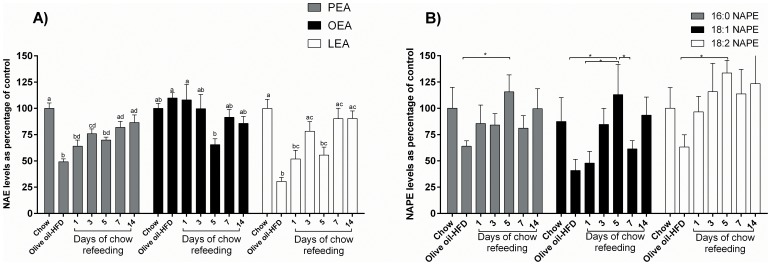
Jejunal levels of anorectic NAEs (PEA, OEA and LEA) and the levels of their corresponding NAPE-precursors (16:0-NAPE, 18:1-NAPE and 18:2-NAPE) in rats fed olive oil-HFD and refed chow. Male Sprague Dawley rats were fed for 7 days with 45E% olive oil-HFD followed by varying length of chow refeeding. Jejunum from the rats were isolated and analyzed for NAEs and NAPEs. Data are expressed as percentage of control. 100% PEA = 886 pmol/g tissue, 100% OEA = 624 pmol/g tissue, 100% LEA = 2754 pmol/g tissue, 100% 16:0-NAPE = 8431 pmol/g tissue, 100% 18:1-NAPE = 3829 pmol/g tissue, 100% 18:2-NAPE = 21723 pmol/g tissue. N = 6–8 animals per group. In A, different letters indicate statistical difference. In B, *p<0.05 1-way ANOVA corrected with Holm Bonferroni test, followed by Fisher's PLSD post hoc test.

Male Sprague Dawley rats were fed one week with an olive oil-HFD (45 E% from fat), and subsequently re-fed with regular chow (14 E% from fat) for up to 2 weeks. Jejunal levels of the anorectic NAEs and NAPEs were measured and the results are shown in figure 1.

Seven days of olive oil-HFD significantly reduced levels of both PEA (51±2.8%) and LEA (70±3.8%) (figure 1A). Gradual restoration of the NAE levels was observed in the rats after they were switched back to chow diet from the olive oil enriched HFD. After 1 day of chow re-feeding, PEA levels were significantly increased compared with the olive oil-HFD group. The PEA level gradually increased; however after 14 days of chow re-feeding the level were still lower compared to the chow control group.

For jejunal LEA levels, three days of chow re-feeding was necessary before the levels were significantly increased compared to the olive oil-HFD group. At this point the levels were furthermore normalized to control levels. Jejunal OEA levels were not changed during the course of the study.

Jejunal NAPE levels tended to follow the same picture as seen for changes in NAE levels with an increase in response to changing the diet from HFD to chow, although significant differences were only seen at a few time points (figure 1B). A positive correlation was observed between the PEA and LEA, respectively and their corresponding precursors (PEA/16:0-NAPE (p = 0.0015); LEA/18:2-NAPE (p = 0.0074)), while this was not seen for OEA/18:1-NAPE (data not shown). This may indicate that the mechanism(s) controlling jejunal levels of anorectic NAEs are generally mediated through regulation of the level of their precursors, the NAPEs.

### Mice fed an olive oil-HFD have reduced jejunal levels of anorectic NAEs

**Figure 2:**
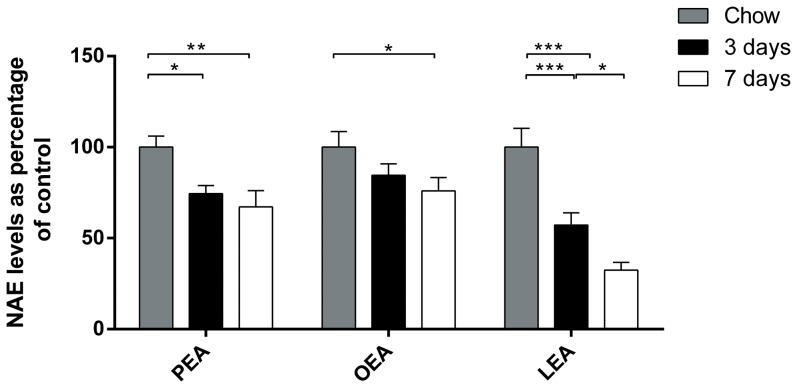
Jejunal levels of anorectic NAEs (PEA, OEA and LEA) in mice fed an olive oil-HFD. Male C57Bl6 mice fed 3 or 7 days of 45E% HFD. Jejunum from the mice were isolated and analyzed for NAEs and NAPEs. Data are expressed as percentage of control. 100% PEA = 712 pmol/g tissue, 100% OEA = 540 pmol/g tissue, 100% LEA = 1826 pmol/g tissue. N = 6–8 animals per group. *p<0.05, **p<0.01, ***p<0.001 1-way ANOVA corrected with Holm Bonferroni test, followed by Fisher's PLSD post hoc test.

In order to investigate whether mice responded in the same way as rats to olive oil-HFD by lowering jejunal levels of anorectic NAEs, mice were fed with olive oil-HFD for either 3 or 7 days. PEA (25±4.4% and 33±8.8%) and LEA (43±6.7% and 68±4.2%) levels were significantly reduced after 3 and 7 days, respectively of feeding olive oil-HFD, whereas OEA level was only significantly reduced after 7 days of dieting (24±7.3%) (figure 2). Thus, mice also decrease their jejunal levels of anorectic NAEs in response to feeding olive oil-HFD.

### Feeding oleic acid to mice resulted in the same decrease in jejunal levels of anorectic NAEs as did feeding of triacylglycerol

**Figure 3:**
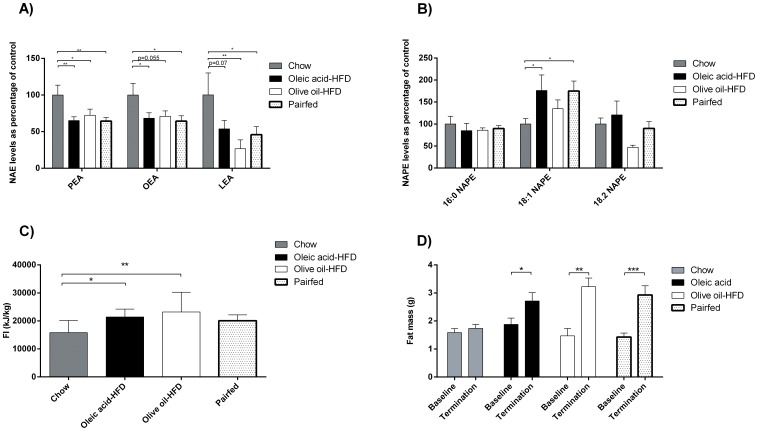
Mice have decreased their jejunal levels of anorectic NAE after oleic acid-HFD to the same extend as when fed olive oil-HFD. Male C57Bl6 mice were fed 7 days with 45E% oleic acid- or olive oil-HFD, respectively. One group receiving olive oil-HFD was also pair fed with the oleic acid-HFD group. Jejunum from the mice were isolated and analyzed for NAEs and NAPEs. A) NAE levels are expressed as percentage of control ± SEM. 100% PEA = 291 pmol/g tissue, 100% OEA = 649 pmol/g tissue, 100% LEA = 1838 pmol/g tissue. B) NAPE levels as percentage of control ± SEM 100% 16∶0 NAPE = 1265 pmol/g tissue, 100% 18.1 NAPE = 473 pmol/g tissue, 100% 18∶2 NAPE = 1531 pmol/g tissue. N = 7–8 animals per group. C) 7 days of accumulated food intake normalized to body weight. D) Body fat mass at baseline and at termination. *p<0.05, **p<0.01, ***p<0.001 1-way ANOVA corrected with Holm Bonferroni test, followed by Fisher's PLSD post hoc test.

In order to investigate, which of the dietary triacylglycerol metabolites (fatty acid or 2-MG), were responsible for regulating jejunal levels of anorectic NAEs, mice were fed 7 days with oleic acid-HFD, olive oil-HFD or chow, respectively. In addition, we included a group fed with olive oil-HFD, but pair-fed to the oleic acid-HFD group. The overall picture observed is that jejunal levels of anorectic NAEs (figure 3A) were reduced when mice were fed oleic acid-HFD, and the levels were reduced to the same extent as during olive oil-HFD. PEA (35±5.2%) and OEA (32±7.5%) levels were significantly reduced, whereas the reductions in LEA did not reach significance. The pair-fed group (fed olive oil-HFD) was included since we speculated that the mice fed the oleic acid-HFD would eat less than the olive oil-HFD. However, this was not the case (figure 3C). NAPE levels (figure 3B) were mostly unchanged between the groups, with the exception of 18∶1 NAPE, which were increased in the oleic acid-HFD group and the pair-fed group compared with chow.

As found in the previous mice study (figure 2), the olive oil-HFD mice had reduced anorectic NAE levels (PEA with 28±8.3%, OEA with 29±7.3% and LEA with 73±12%, Figure 3A). Since the caloric density was higher in the HFDs, more calories were consumed by the groups of mice maintained on the HFD (figure 3C). The pair-fed group had the same intake as the oleic acid-HFD group.

As anticipated, the respiratory exchange ratio (RER) (Table 1) was higher in the dark phase since mice are active during the dark period. When more fat were available to the mice, RER were lowered, indicative of a switch from carbohydrate as substrate for metabolism to using triacylglycerol/oleic acid as the substrate.

**Table 1:** **Metabolic parameters in the mice fed the olive oil- or oleic acid-HFD, respectively.**

	RER		vO2 (ml/h/kg)	
	*Light*	*Dark*	*Light*	*Dark*
**Chow**	0.90±0.005***	0.97±0.008	3556±107***	4399±99
**Olive oil-HFD**	0.86±0.008# *	0.88±0.004##	4557±322#	5272±404##
**Oleic acid-HFD**	0.85±0.007# *	0.88±0.006##	3640±193¤	4278±195¤¤
**Pair-fed**	0.86±0.01#	0.88±0.005##	3326±103***	3882±98

# p<0.05 vs. Chow light phase.

## p<0.05 vs. Chow dark phase.

*P<0.05 between light and dark phase in mice on the same diet.

***p<0.0001 between light and dark phase in mice on the same diet.

¤ p<0.01 vs. olive oil-HFD light phase.

¤¤ p<0.01 vs. olive oil-HFD dark phase.

Furthermore, fat mass was significantly increased in the oleic acid-HFD, olive oil-HFD and the pair-fed groups (Figure 3D).

## Discussion

Nutrient sensing in the gastrointestinal system mediates many biological functions including appetite regulation [33], [34]. After initial sensing, the signal can be transmitted via various mechanisms, e.g. by releasing intestinal hormones and/or activation of vagal nerves. Jejunal levels of anorectic NAEs may be involved in mediating such sensing as evidence indicate that both exogenous and endogenous anorectic NAEs may inhibit food intake probably via the vagus nerve [3], [8], [10], [11], [35]. Recently, we have shown that feeding rats an olive oil-HFD (1 to 7 days) reduces jejunal levels of anorectic NAEs in a dose and time-dependent manner [9]. In that study, OEA decreased in response to olive oil, while in the present refeeding study we do not see the same reductions. One possible explanation for why OEA levels are not reduced could be that the animals were fed an oleic acid-rich HFD, which could have masked/counteracted the effects of HFD, since the gastrointestinal system was overloaded with the fatty acid precursor oleic acid, as discussed previously [11]. However, the levels of the two other anorectic NAEs, PEA and LEA were significantly reduced. The current study show that reductions of the anorectic NAE levels, in response to 7 days of olive oil-HFD can be reversed by switching the diet back to regular chow. This effect became significant after 3 days of chow feeding. The apparent lower NAE levels at day 5 of refeeding may be due to biological variations within the group of refed mice. This rapid regulation indicates that jejunal levels of anorectic NAEs could have a biological effect as sensor for the dietary fat status. The reductions jejunal levels of anorectic NAEs (OEA and PEA, LEA was not measured) in response to HFD has also been observed after 8 weeks of HFD, however this reduction was not seen after 14 weeks of HFD [36].

The HFD-induced reduction of NAPE levels were also reversed, with significance increase after 5 days of chow feeding of the rats. Correlating the *N*-acyl-group specific NAE and NAPE levels from the rat study shows a positive correlation between the two parameters for most *N*-acyl-group species (data not shown). These results, together with the results from Petersen *et al* [13] and Schwartz *et al* [37], suggest that jejunal levels of anorectic NAEs are regulated upstream of NAPE formation, probably through regulation of the activity of the *N*-acyltransferease [19], the enzyme responsible for generating NAPE. No other intestinal segments were analyzed, since we previously have shown that NAE levels only changes in the jejunum followed HFD feeding [14]. We also found that HFD decreased jejunal levels of the anorectic NAEs in mice and this seems to be supported in a recent study where they measured OEA levels in small intestine in mice on a HFD [38]. The starving-refeeding response in rats [8], [13] on jejunal levels of anorectic NAEs has also been seen in mice [39]. These findings demonstrate, as would have been anticipated, that reductions of jejunal levels of anorectic NAEs are a more general phenomenon, which translate across species.

The observation that dietary fat decreased jejunal levels of anorectic NAEs in both rats and mice may suggest that this may also happen in humans. In both humans [29] and experimental rodents [40] voluntary intake of HFD may induce overconsumption of calories and thereby promote obesity. A reduced jejunal level of anorectic NAEs may contribute to this over-consumption. Recently, Tellez *et al* [38] have found a reduced dopamine release in the dorsal striatum in response to gastrointestinal fat infusion. This dopamine deficiency was normalized by intestinal infusion of OEA, pointing to an important role of endogenous OEA (and probably also LEA and PEA) as homeostatic signals, which dictate the amount of dietary fat to be ingested [38].

Dietary triacylglycerol is upon digestion hydrolyzed, by various lipases in the upper part of the gastrointestinal tract, mainly to 2-MG and fatty acids. GPR119, which is located in GLP-1-releasing intestinal L-cells, has recently been shown to be activated by 2-oleoyl glycerol [21], [22] thereby being one of several fat sensors in the gastrointestinal system. Several receptors have been shown to be involved in fatty acid sensing [41], including the receptors GPR40 [23], [24], [42], GPR120 [25] and the transporter CD36 [26]. Intestinal fatty acid beta-oxidation [43], intestinal protein kinase C isoenzymes [44], [45] and bile acid receptor TGR5 [46] may also contribute to sensing of dietary fat in the intestine.

In our quest to sort out how dietary fat and anorectic NAEs regulate food intake, we wished to identify the dietary fat metabolite, which are involved in down-regulation of anorectic NAEs in the jejunum following HFD. Mice were fed either an olive oil-based HFD or the corresponding fatty acid, oleic acid-based HFD. We have previously shown that other types of dietary triacylglycerol also induces reduction of intestinal NAE levels, but in this study, we only tested the triacylglycerol/fatty acid pair olive oil/oleic acid, since it is believed that the other triacylglycerol/fatty acid pairs will act similar to the olive oil/oleic acid induced reductions. After 7 days of feeding, PEA and OEA levels in the oleic acid-HFD fed group were reduced to the same extent compared to the olive oil-HFD. Reductions in LEA levels did not reach statistical significance even though the trend is obvious. Both groups of mice (olive oil- and oleic acid-HFD) significantly increased caloric intake and their fat depot, indicating that the oleic acid-HFD fed mice were able to absorb and store excess energy even without dietary availability of 2-MG. The results clearly show that dietary non-esterified fatty acids are involved in the regulation of jejunal levels of anorectic NAEs, which strongly suggests that GPR119 is not involved in mediating the effect of HFD on jejunal levels of anorectic NAEs. Thus, in future studies we will focus on GPR40, GPR120, CD36, protein kinase C isoenzymes, intestinal beta-oxidation and TGR5 as one of these may be the sensor that is involved in the down-regulation of jejunal levels of anorectic NAEs.

In summary, we have in this study shown that down regulation of anorectic NAE levels in the jejunum of rats after HFD is reversible when changing the diet back to regular chow. Furthermore, we have shown that two different species (mouse and rats) both responded to dietary fat by lowering NAE levels, and that the olive oil-metabolite oleic acid was sufficient to down-regulate NAE levels in mice. In rats, the decrease in NAE levels may be caused by a concomitant decrease in NAPE levels, while such a mechanism is less clear in mice. The reduced intestinal NAE levels may possibly contribute to an increased energy intake and thereby to development of obesity. The sensing mechanisms contributing to the decreased NAE levels in the intestine involve sensing of free fatty acids.

## References

[1] Hansen HS (2010) Palmitoylethanolamide and other anandamide congeners. Proposed role in the diseased brain. Exp Neurol 224: 48–55. 20353771 10.1016/j.expneurol.2010.03.022

[2] Hansen HS, Diep TA (2009) N-acylethanolamines, anandamide and food intake. Biochem Pharmacol 78: 553–560. 19413995 10.1016/j.bcp.2009.04.024

[3] Piomelli D (2013) A fatty gut feeling. Trends Endocrinol Metab 24: 332–341. 23567058 10.1016/j.tem.2013.03.001 PMC3778443

[4] Calignano A, La Rana G, Giuffrida A, Piomelli D (1998) Control of pain initiation by endogenous cannabinoids. Nature 394: 277–281. 9685157 10.1038/28393

[5] Jaggar SI, Sellaturay S, Rice ASC (1998) The endogenous cannabinoid anandamide, but not the CB2 ligand palmitoylethanolamide, prevents the viscero-visceral hyperreflexia associated with inflammation of the rat urinary bladder. Neurosci Lett 253: 123–126. 9774165 10.1016/s0304-3940(98)00621-1

[6] Solorzano C, Zhu C, Battista N, Astarita G, Lodola A, et al (2009) Selective N-acylethanolamine-hydrolyzing acid amidase inhibition reveals a key role for endogenous palmitoylethanolamide in inflammation. Proc Natl Acad Sci U S A 106: 20966–20971. 19926854 10.1073/pnas.0907417106 PMC2791595

[7] Mazzari S, Canella R, Petrelli L, Marcolongo G, Leon A (1996) *N*-(2-Hydroxyethyl)hexadecanamide is orally active in reducing edema formation and inflammatory hyperalgesia by down-modulating mast cell activation. Eur J Pharmacol 300: 227–236. 8739213 10.1016/0014-2999(96)00015-5

[8] Rodríguez de Fonseca F, Navarro M, Gómez R, Escuredo L, Nava F, et al (2001) An anorexic lipid mediator regulated by feeding. Nature 414: 209–212. 11700558 10.1038/35102582

[9] Diep TA, Madsen AN, Holst B, Kristiansen MM, Wellner N, et al (2011) Dietary fat decreases intestinal levels of the anorectic lipids through a fat sensor. FASEB J 25: 765–774. 20959516 10.1096/fj.10-166595

[10] Fu J, Kim J, Oveisi F, Astarita G, Piomelli D (2008) Targeted enhancement of oleoylethanolamide production in proximal small intestine induces across-meal satiety in rats. Am J Physiol Regul Integr Comp Physiol 295: R45–R50. 18434444 10.1152/ajpregu.00126.2008 PMC2494809

[11] Hansen HS (2014) Role of anorectic N-acylethanolamines in intestinal physiology and satiety control with respect to dietary fat. Pharmacol Res. S1043-6618(14)00029-2 [pii];10.1016/j.phrs.2014.03.006 [doi]. 10.1016/j.phrs.2014.03.006 24681513

[12] Fu J, Gaetani S, Oveisi F, LoVerme J, Serrano A, et al (2003) Oleoylethanolamide regulates feeding and body weight through activation of the nuclear receptor PPARα. Nature 425: 90–93. 12955147 10.1038/nature01921

[13] Petersen G, Sorensen C, Schmid PC, Artmann A, Tang-Christensen M, et al (2006) Intestinal levels of anandamide and oleoylethanolamide in food-deprived rats are regulated through their precursors. Biochim Biophys Acta Mol Cell Biol Lipids 1761: 143–150. 10.1016/j.bbalip.2005.12.011 16478679

[14] Artmann A, Petersen G, Hellgren LI, Boberg J, Skonberg C, et al (2008) Influence of dietary fatty acids on endocannbinoid and n-acylethanolamine levels in rat brain, liver and small intestine. Biochim Biophys Acta Mol Cell Biol Lipids 1781: 200–212. 10.1016/j.bbalip.2008.01.006 18316044

[15] Gaetani S, Oveisi F, Piomelli D (2003) Modulation of meal pattern in the rat by anorexic lipid mediator oleoylethanolamide. Neuropsychopharmacology 28: 1311–1316. 12700681 10.1038/sj.npp.1300166

[16] Okamoto Y, Morishita J, Tsuboi K, Tonai T, Ueda N (2004) Molecular characterization of a phospholipase D generating anandamide and its congeners. J Biol Chem 279: 5298–5305. 14634025 10.1074/jbc.M306642200

[17] Wellner N, Diep TA, Janfelt C, Hansen HS (2013) N-acylation of phosphatidylethanolamine and its biological functions in mammals. Biochim Biophys Acta 1831: 652–662. 23000428 10.1016/j.bbalip.2012.08.019

[18] Gillum MP, Zhang D, Zhang XM, Erion DM, Jamison RA, et al (2008) N-acylphosphatidylethanolamine, a gut- derived circulating factor induced by fat ingestion, inhibits food intake. Cell 135: 813–824. 19041747 10.1016/j.cell.2008.10.043 PMC2643061

[19] Wellner N, Tsuboi K, Madsen AN, Holst B, Diep TA, et al (2011) Studies on the anorectic effect of N-acylphosphatidylethanolamine and phosphatidylethanolamine in mice. Biochim Biophys Acta 1811: 508–512. 21723414 10.1016/j.bbalip.2011.06.020

[20] Kleberg K, Hassing HA, Hansen HS (2014) Classical endocannabinoid-like compounds and their regulation by nutrients. BioFactors. 10.1002/biof.1158 [doi]. 10.1002/biof.1158 24677570

[21] Hansen HS, Rosenkilde MM, Holst JJ, Schwartz TW (2012) GPR119 as a fat sensor. Trends Pharmacol Sci 33: 374–381. 22560300 10.1016/j.tips.2012.03.014

[22] Hansen KB, Rosenkilde MM, Knop FK, Wellner N, Diep TA, et al (2011) 2-Oleoyl Glycerol Is a GPR119 Agonist and Signals GLP-1 Release in Humans. J Clin Endocrinol Metab 96: E1409–E1417. 21778222 10.1210/jc.2011-0647

[23] Itoh Y, Kawamata Y, Harada M, Kobayashi M, Fujii R, et al (2003) Free fatty acids regulate insulin secretion from pancreatic β cells through GPR40. Nature 422: 173–176. 12629551 10.1038/nature01478

[24] Briscoe CP, Tadayyon M, Andrews JL, Benson WG, Chambers JK, et al (2003) The orphan G protein-coupled receptor GPR40 is activated by medium and long chain fatty acids. J Biol Chem 278: 11303–11311. 12496284 10.1074/jbc.M211495200

[25] Hirasawa A, Tsumaya K, Awaji T, Katsuma S, Adachi T, et al (2005) Free fatty acids regulate gut incretin glucagon-like peptide-1 secretion through GPR120. Nature Med 11: 90–94. 15619630 10.1038/nm1168

[26] Naville D, Duchampt A, Vigier M, Oursel D, Lessire R, et al (2012) Link between Intestinal CD36 Ligand Binding and Satiety Induced by a High Protein Diet in Mice. PLoS ONE 7: e30686. 22295104 10.1371/journal.pone.0030686 PMC3266275

[27] Madsen AN, Hansen G, Paulsen S, Lykkegaard K, Tang-Christensen M, et al (2010) Long-term characterization of the diet-induced obese and diet resistant rat model: A polygenetic rat model mimicking the human obesity syndrome. J Endocrinol 206: 287–296. 20508079 10.1677/JOE-10-0004

[28] Judge MK, Zhang J, Tumer N, Carter C, Daniels MJ, et al (2008) Prolonged hyperphagia with high-fat feeding contributes to exacerbated weight gain in rats with adult-onset obesity. Am J Physiol Regul Integr Comp Physiol 295: R773–R780. 18596107 10.1152/ajpregu.00727.2007 PMC2536857

[29] Prentice AM (1998) Manipulation of dietary fat and energy density and subsequent effects on substrate flux and food intake. Am J Clin Nutr 67: 535S–541S. 9497166 10.1093/ajcn/67.3.535S

[30] Skonberg C, Artmann A, Cornett C, Hansen SH, Hansen HS (2010) Pitfalls in the sample preparation and analysis of N-acylethanolamines. J Lipid Res 51: 3062–3073. 20447930 10.1194/jlr.D004606 PMC2936757

[31] Petersen G, Chapman KD, Hansen HS (2000) A rapid phospholipase D assay using zirconium precipitation of anionic substrate phospholipids: application to *N*-acylethanolamine formation *in vitro* . J Lipid Res 41: 1532–1538. 10974061

[32] Schmid PC, Natarajan V, Weis BK, Schmid HHO (1986) Hydrolysis of *N*-acylated glycerophospholipids by phospholipase A_2_ and D: a method of identification and analysis. Chem Phys Lipids 41: 195–207. 3815621 10.1016/0009-3084(86)90022-8

[33] Reimann F, Tolhurst G, Gribble FM (2012) G-protein-coupled receptors in intestinal chemosensation. Cell Metab 15: 421–431. 22482725 10.1016/j.cmet.2011.12.019

[34] Sclafani A, Ackroff K (2012) Invited Review: The role of gut nutrient sensing in stimulating appetite and conditioning food preferences. Am J Physiol Regul Integr Comp Physiol 302: R119–R133. 10.1152/ajpregu.00038.2012 PMC3362145 22442194

[35] Nielsen MJ, Petersen G, Astrup A, Hansen HS (2004) Food intake is inhibited by oral oleoylethanolamide. J Lipid Res 45: 1027–1029. 15060091 10.1194/jlr.C300008-JLR200

[36] Aviello G, Matias I, Capasso R, Petrosino S, Borrelli F, et al (2008) Inhibitory effect of the anorexic compound oleoylethanolamide on gastric emptying in control and overweight mice. J Mol Med 86: 413–422. 18278475 10.1007/s00109-008-0305-7

[37] Schwartz GJ, Fu J, Astarita G, Li X, Gaetani S, et al (2008) The lipid messenger OEA links dietary fat intake to satiety. Cell Metab 8: 281–288. 18840358 10.1016/j.cmet.2008.08.005 PMC2572640

[38] Tellez LA, Medina S, Han W, Ferreira JG, Licona-Limon P, et al (2013) A gut lipid messenger links excess dietary fat to dopamine deficiency. Science 341: 800–802. 23950538 10.1126/science.1239275

[39] Syed SK, Bui HH, Beavers LS, Farb TB, Ficorilli J, et al (2012) Regulation of GPR119 receptor activity with endocannabinoid - like lipids. Am J Physiol Endocrinol Metab 303: E1469–E1478. 23074242 10.1152/ajpendo.00269.2012

[40] Rothwell NJ, Stock MJ, Warwick BP (1983) The effect of high fat and high carbohydrate cafeteria diets on diet-induced thermogenesis in the rat. Int J Obes 7: 263–270. 6684102

[41] Rasoamanana R, Darcel N, Fromentin G, Tome D (2012) Nutrient sensing and signalling by the gut. Proc Nutr Soc 1–10. 10.1017/S0029665112000110 22453062

[42] Liou AP, Lu X, Sei Y, Zhao X, Pechhold S, et al (2011) The G-Protein-Coupled Receptor GPR40 Directly Mediates Long-Chain Fatty Acid-Induced Secretion of Cholecystokinin. Gastroenterology 140: 903–912. 20955703 10.1053/j.gastro.2010.10.012 PMC4717904

[43] Langhans W, Leitner C, Arnold M (2011) Dietary fat sensing via fatty acid oxidation in enterocytes: possible role in the control of eating. Am J Physiol Regul Integr Comp Physiol 300: R554–R565. 21148477 10.1152/ajpregu.00610.2010

[44] Iakoubov R, Ahmed A, Lauffer LM, Bazinet RP, Brubaker PL (2011) Essential Role for Protein Kinase C{zeta} in Oleic Acid-Induced Glucagon-Like Peptide-1 Secretion in Vivo in the Rat. Endocrinology 152: 1244–1252. 21325047 10.1210/en.2010-1352

[45] Rasmussen BA, Breen DM, Lam TK (2012) Lipid sensing in the gut, brain and liver. Trends Endocrinol Metab 23: 49–55. 22169756 10.1016/j.tem.2011.11.001

[46] Thomas C, Gioiello A, Noriega L, Strehle A, Oury J, et al (2009) TGR5-mediated bile acid sensing controls glucose homeostasis. Cell Metab 10: 167–177. 19723493 10.1016/j.cmet.2009.08.001 PMC2739652

